# Latent profile analysis of nurses’ the quality of palliative nursing care

**DOI:** 10.3389/fmed.2025.1537851

**Published:** 2025-05-09

**Authors:** Xue Dong, Yan Liu, Kui Fang, Zezhou Wang

**Affiliations:** Department of Neurosurgery, The First Affiliated Hospital of China Medical University, Shenyang, China

**Keywords:** nurses, palliative care, latent profile analysis, accompaniment, quality palliative care

## Abstract

**Objectives:**

To explore the status of nurses’ the quality of palliative nursing care, analyze the potential characteristics and influencing factors of different types of nurses’ the quality of palliative nursing care, and provide reference for formulating intervention programs of nurses’ the quality of palliative nursing care.

**Methods:**

A total of 316 nurses from three hospitals in Liaoning Province were selected by using convenience sampling method in April 2024. General information questionnaire, the Palliative Nursing Care Quality Scale (PNCQS), Meaning in Life Questionnaire, the Chinese version of the Bradley Attitude Assessment Questionnaire, and the Coping with Death Scale Short Version were used to investigate. SPSS 26.0 and Mplus 8.3 were used for data analysis.

**Results:**

The score of the quality of palliative nursing care was (3.76 ± 0.77), and two latent profiles were identified: the low-quality group and the high-quality group, accounting for 47% and 53%. Attitude of nurses toward palliative care, meaning in life, and coping with death competence were the factors influencing the potential categories of nurses’ the quality of palliative nursing care (*P* < 0.05).

**Conclusion:**

Nurses’ the quality of palliative nursing care was in the medium level, and there were two potential categories: the low palliative nursing care quality group and the high palliative nursing care quality group. Nursing managers should take precise measures according to different potential categories of influencing factors to improve the quality of palliative nursing care.

## 1 Introduction

Palliative care aims to provide caring services through comprehensive medical and emotional support to help patients achieve comfort and dignity in the final stages of life. The quality of palliative nursing care is an important index to measure the quality of death and an important condition to ensure the sustainable development of palliative care (1). A study showed that nurses assume various roles in the palliative care process, such as professional caregiver and multidisciplinary team coordinator (2). Nurses are the main providers of palliative care and the core force for promoting the development of palliative care in Chinese. Accompaniment can enable patients to have a fulfilling and high-quality life experience, thereby improving their psychological state and happiness. The quality of palliative nursing care directly affects the quality of life of the patients and their families. The quality assessment of palliative nursing care and the exploration of influencing factors can help managers identify quality deficiencies and provide valuable insights for future strategy development. The Palliative Nursing Care Quality Scale (PNCQS), developed by Zulueta-Egea in 2020 (3), was designed and validated by nurses working in palliative care. The scale was introduced to China by Hao (4) in October 2023 and has good reliability and validity. Latent profile analysis (LPA) is an individual-centered classification statistical method, which can judge the potential characteristics of individuals according to their response patterns on explicit variables, and include individuals with similar characteristics into the same category and calculate the category distribution probability (5) to provide an effective basis for accurate intervention. In China, there have been no studies on categories or groups of nurses’ the quality of palliative nursing care obtained by potential profile analysis. In this study, LPA was used to study nurses’ the quality of palliative nursing care and explore the characteristics of each classification, aiming to provide reference for formulating the intervention plan of nurses’ the quality of palliative nursing care.

## 2 Materials and methods

### 2.1 Participants

A convenience method was used to the qualified clinical nurses in three hospitals in Shenyang in April 2024. The inclusion criteria were (1) possession of a nurse practitioner’s license; (2) More than 1 year of working time; (3) informed consent and voluntary participation in this survey. The exclusion criteria were nurses on maternity leave, sick leave, internship or advanced training. As previous research suggested a minimum sample size of 300–500 for LPA studies (6), a total of 316 nurses were involved in this study.

### 2.2 Procedures

Contact the head nurses in each ward of the hospital, inform them of the purpose of the study, and investigate after obtaining consent. The head nurse sent the QR code of the questionnaire star to the nurse to scan the code and fill in. Each question was set as a required answer. Each account could only be filled in once, and the filling time was 8–10 min.

### 2.3 Measures

#### 2.3.1 Socio-demographic questionnaire

Self-designed after a preliminary review of the literature. These include gender, nationality, religious faith, age, marital status, education, job title, department position, current department, income per month, working time in clinical nursing (years), whether you are specialist nurses or not, familiarity with palliative knowledge, years of palliative contact, number of terminal patients cared for in the last 6 months, whether you have received or participated in courses/training related to palliative care, whether you have the experience in caring for the terminal family member, whether you have discussed death with the patient, whether you have discussed death with the patient’s family, self-assessment of palliative work competence.

#### 2.3.2 Palliative Nursing Care Quality Scale (PNCQS)

Palliative nursing care quality scale developed by Zulueta Egea (3) and Chineseized by Hao (4) was used to assess nurses’ the quality of palliative nursing care. The scale has five dimensions, which are control and relief of symptoms (three items), family and/or main caregiver (five items), therapeutic relationships with the patient and family (five items), spiritual support (five items), and continuity of care (two items), and has 20 items. The scale was based on a five-point scale with values from 1 (almost never) to 5 (almost always), with a total score ranging from 20 to 100 points and higher scores indicating the higher perceived quality of palliative nursing care. The scale yielded good internal reliability, with a Cronbach’s α of 0.94.

#### 2.3.3 Bradley Attitude Assessment Questionnaire

Bradley Attitude Assessment Questionnaire developed by Yale University School of Medicine (7) and Chineseized by Zhou Min (8) was used to assess the attitude of nurses toward palliative care. The scale has three dimensions, which are professional responsibilities and roles (four items), hospice care effectiveness (five items), and nurse-patient communication (three items), and has 12 items. The scale was based on a five-point scale with values from 1 (strongly disagree) to 5 (strongly agree), with a total score ranging from 12 to 60 points and higher scores indicating the better attitudes of nurses toward palliative care. In this study, the Cronbach’s α value was 0.89.

#### 2.3.4 Meaning in Life Questionnaire

Meaning in Life Questionnaire was developed by Steger (9) and revised by Gan Yiqun (10). The scale has two dimensions, which are sense of existential meaning (five items) and sense of seeking meaning (four items), and has nine items. The scale was based on a seven-point scale with values from 1 (strongly disagree) to 7 (strongly agree), with a total score ranging from 9 to 63 points and higher scores indicating the higher level of meaning in life. The scale yielded good internal reliability, with a Cronbach’s α of 0.83.

#### 2.3.5 The Coping with Death Scale Short Version

The Coping with Death Scale Short Version developed by Oliver (11) and Chineseized by Qian Li (12) was used to assess coping with death competence. The scale is a single dimension scale and has nine items. The scale was based on a seven-point scale with values from 1 (strongly disagree) to 7 (strongly agree), with a total score ranging from 9 to 63 points and higher scores indicating the higher coping with death competence. In this study, the Cronbach’s α value was 0.88.

### 2.4 Statistical analysis

In this study, Mplus7.0 was used for latent profile analysis, and profiles from 1 to 4 were sequentially selected for fitting with the score of five dimensions of PNCQS as the exogenous variable. Akaike Information Criterion (AIC), Bayesian Information Criterion (BIC), and adjusted Bayesian Information Criterion (BIC) were used to judge the model fit, the smaller values of the three metrics indicated better model fit; Entropy is between 0 and 1, with larger values indicating better model fit (= 0.8 corresponds to 90% of cases correctly classified) (13). The fit differences among the models were compared by Bootstrap Likelihood Ratio Test (BLRT) and lo-Mendell-Rubin (LMR), and the difference was statistically significant (*P* < 0.05) indicated that the k-class model was superior to a model with k-1 classes. SPSS 25.0 software (14) was used for data analysis. The measurement data conforming to the normal distribution were described as means ± standard deviation (x ± s). Comparisons between groups were made employing the chi-square test and *t*-test. The factors with significant differences in the results of the univariate analysis were used as independent variables, and the results of the latent profiles were used as the dependent variables to conduct binary Logistic regression. The test level was α = 0.05 (two-tailed).

### 2.5 Ethical considerations

All participants were informed of the purpose, risks and benefits of the study before completing the questionnaire. Our research was in accordance with the Declaration of Helsinki (15) and was approved by the ethics committee of the first Affiliated Hospital of China Medical University (AF-SOP-07-1.2-01).

## 3 Results

### 3.1 Socio-demographic characteristics of the participants

Among the 316 nurses, 31 cases (9.81%) were male and 285 (90.19%) were female; age: 60 cases (18.99%) ≤ 25 years old, 96 cases (30.38%) 26–35 years old, 160 cases (50.63%) ≥ 36 years old; Table 1 displays all of the remaining general data. The PNCQS average score of nurses was (3.76 ± 0.77). The total score of the Bradley Attitude Assessment Questionnaire was (37.87 ± 3.64) points, the average score was (3.16 ± 0.30) points. The total score of the Meaning in Life Questionnaire was (45.10 ± 8.83) points, the average score was (5.01 ± 0.98) points (See Table 2).

**TABLE 1:** Comparison of the distribution of potential profiles of nurses’ the quality of palliative nursing care with characteristics (*n* = 316).

Variables		Number	The low- quality group	The high-quality group	χ^2^/F	*P*
Gender	Male	31	14	17	0.039	0.844
	Female	285	134	151	–	–
Nationality	Han	265	123	142	0.117	0.733
	Else	51	25	26	–	–
Religious faith	Yes	10	9	1	6.041	0.014
	No	306	139	167	–	–
Age	≤ 25	60	30	30	1.507	0.471
	26–35	96	40	56	–	–
	≥ 36	160	78	82	–	–
Marital status	Married	202	90	112	2.200	0.333
	Single	103	54	49	–	–
	Else	11	4	7	–	–
Education	Below university and college	21	14	7	3.556	0.169
	Undergraduate	282	128	154	–	–
	Master’s degree and above	13	6	7	–	–
Job title	Junior nurse	54	27	27	5.811	0.121
	Senior nurse	115	45	70	–	–
	Nurse-in-charge	131	70	61	–	–
	Vice-director nurse and above	16	6	10	–	–
Department position	Ordinary nurse	254	115	139	1.523	0.677
	Team leader	22	12	10	–	–
	Head nurse and above	12	7	5	–	–
	Else	28	14	14	–	–
Current department	Oncology department	86	42	44	6.231	0.284
	Department of geriatrics	14	10	4	–	–
	Intensive care unit	74	29	45	–	–
	Internal medicine department	73	37	36	–	–
	Surgery department	48	20	28	–	–
	Else	21	10	11	–	–
Income per month (yuan)	< 2,000	12	4	8	1.498	0.683
	2,000–5,000	47	20	27	–	–
	5,000–10,000	141	67	74	–	–
	> 10,000	116	57	59	–	–
Working time in clinical nursing (years)	≤5	90	41	49	1.192	0.755
	6–10	31	12	19	–	–
	11–20	155	75	80	–	–
	≥ 21	40	20	20	–	–
Whether you are specialist nurses or not	Yes	119	60	59	0.985	0.321
	No	197	88	109	–	–
Familiarity with palliative knowledge	Familiarity is low	96	46	50	–	–
	General familiarity	183	86	97	0.536	0.911
	Quite familiar	26	12	14	–	–
	Very familiar	11	4	7	–	–
Years of palliative contact	< 1	220	104	116	1.539	0.463
	1–5	73	36	37	–	–
	> 5	23	8	15	–	–
Number of terminal patients cared for in the last 6 months	0	145	72	73	1.058	0.589
	1–10	158	71	87	–	–
	≥ 11	13	5	8	–	–
Whether you have received or participated in courses/training related to palliative care	Yes	68	24	44	4.635	0.031
	No	248	124	124	–	–
Whether you have the experience in caring for the terminal family member	No	183	89	94	0.565	0.452
	Yes	133	59	74	–	–
Whether you have discussed death with the patient	Yes	105	39	66	5.933	0.015
	No	211	109	102	–	–
Whether you have discussed death with the patient’s family	Yes	129	47	82	9.471	0.002
	No	187	101	86		
Self-assessment of palliative work competence	Cannot	46	27	19	13.037	0.001
	Generally	195	99	96	–	–
	Can	75	22	53	–	–
Bradley Attitude Assessment Questionnaire	37.87 ± 3.640		36.71 ± 2.853	38.90 ± 3.945	–5.586	0.000
Meaning in Life Questionnaire	45.10 ± 8.834		40.04 ± 7.338	49.55 ± 7.560	–11.338	0.000
The Coping With Death Scale Short Version	41.91 ± 9.488		37.62 ± 6.888	45.70 ± 9.864	–8.329	0.000

**TABLE 2:** Scores of nurses’ the quality of palliative nursing care, attitude of nurses toward palliative care, and meaning in life (*n* = 316).

Variables	Number of items	Total score (x ± s)	Average score (x ± s)
Palliative nursing care quality	20	75.24 ± 15.38	3.76 ± 0.77
Control and relief of symptoms	3	11.62 ± 2.44	3.87 ± 0.81
Family and/or main caregiver	5	18.65 ± 3.98	3.73 ± 0.80
Therapeutic relationships with the patient and family	5	19.13 ± 4.04	3.83 ± 0.81
Spiritual support	5	18.50 ± 4.06	3.70 ± 0.81
Continuity of care	2	7.35 ± 1.83	3.68 ± 0.92
Bradley Attitude Assessment	12	37.87 ± 3.64	3.16 ± 0.30
Professional responsibilities and roles	4	13.34 ± 2.90	3.34 ± 0.73
Hospice care effectiveness	5	16.21 ± 2.08	3.24 ± 0.41
Nurse-patient communication	3	8.32 ± 1.50	2.77 ± 0.50
Meaning in Life	9	45.10 ± 8.83	5.01 ± 0.98
Sense of seeking meaning	4	19.42 ± 4.82	4.85 ± 1.21
Sense of existential meaning	5	25.68 ± 5.30	5.14 ± 1.06

### 3.2 Latent profiles analysis of PNCQS

**FIGURE 1:**
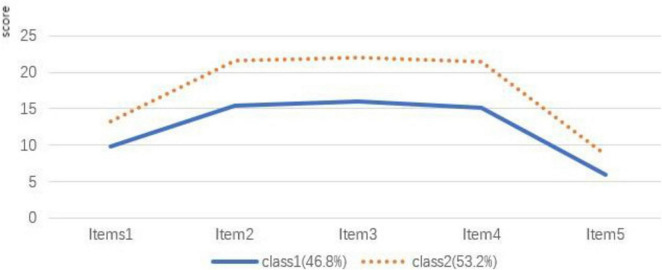
Response to each items in the two potential profiles of nurses’ the quality of palliative nursing care with characteristics (*n* = 316).

Using the five dimensions of the PNCQS as exogenous indicators, individual-centered potential profile analysis of nurses’ the quality of palliative nursing care was performed and latent profiles models for categories 1–4 were fitted sequentially, as shown in Table 3. The AIC, BIC, and aBIC kept decreasing with the increase if the profiles, and when the number of categories was two, the entropy value was 0.896, and the LMR and BLRT values were statistically significant (*p* < 0.05); when the number of categories was three, the LMR and BLRT values were not statistically significant (*p* > 0.05), so two profiles were selected on a comprehensive basis. On the basis of the determination of the latent profile model, a chart was drawn based on scores of the two categories of nurses’ the quality of palliative nursing care on the five dimensions (see Figure 1). Compared with Class 1, Class 2 had higher scores in all dimensions, so it was named “the high palliative nursing care quality group,” accounting for 53% of the total. Category 1 had low scores in all dimensions, so it was named “the low palliative nursing care quality group,” accounting for 47% of the total.

**TABLE 3:** Model fit indices of latent profile analysis (*n* = 316).

Latent profile	AIC	BIC	aBIC	Entropy	LMR (*p*-value)	BLRT (*p-*value)	Latent class probability
1-profile	8087.341	8124.898	8093.181	–	–	–	–
2-profile	7165.024	7225.116	7174.368	0.896	0.0018	0.0015	0.468/0.532
3-profile	6475.919	6558.546	6488.767	0.967	0.1424	0.137	0.361/0.424/0.215
4-profile	6053.232	6158.393	6069.584	0.982	0.0609	0.0572	0.025/0.348/0.408/0.219

### 3.3 Distributional characteristics of latent profiles of nurses’ the quality of palliative nursing care

The results of the univariate analysis showed that the differences in the distribution of the latent profiles of nurses’ the quality of palliative nursing care of religious faith, whether you have received or participated in courses/training related to palliative care, whether you have discussed death with the patient, whether you have discussed death with the patient’s family, self-assessment of palliative work competence, attitude of nurses toward palliative care, meaning in life, and coping with death competence were statistically significant (*p* < 0.05), as shown in Table 1.

### 3.4 A multifactorial analysis of factors influencing latent profiles of nurses’ the quality of palliative nursing care

The two potential categories of nurses’ the quality of palliative nursing care were taken as dependent variables, and the statistically significant indicators in the univariate analysis were taken as independent variables for logistic regression analysis. The assignment of independent variables was shown in Table 4. The results of multiple logistic regression analysis showed that attitude of nurses toward palliative care (OR = 1.199, 95% CI = 1.093–1.316, *p* < 0.01), meaning in life (OR = 1.146, 95% CI = 1.097–1.197, *p* < 0.01), and coping with death competence (OR = 1.052, 95% CI = 1.010–1.096, *p* < 0.05) were influential factors affecting the potential profile of nurses’ the quality of palliative nursing care.

**TABLE 4:** The multifactor analysis of nurses’ the quality of palliative nursing care by logistic regression.

Variable	β	SE	Wald Chi-square	*P*	OR	95% CI
Constant term	–18.029	3.280	30.219	0.000	0.000	–
Religious faith	1.866	1.164	2.569	0.109	6.460	0.660–63.225
Whether you have received or participated in courses/training related to palliative care	–0.125	0.371	0.114	0.736	0.882	0.426–1.827
Whether you have discussed death with the patient	–0.272	0.358	0.580	0.446	0.762	0.378–1.535
Whether you have discussed death with the patient’s family	–0.196	0.326	0.361	0.548	0.822	0.434–1.558
Self-assessment of palliative work competence	0.222	0.256	0.751	0.386	1.249	0.756–2.063
Bradley Attitude Assessment Questionnaire	0.182	0.047	14.689	0.000	1.199	1.093–1.316
Meaning in Life Questionnaire	0.136	0.022	37.629	.000	1.146	1.097–1.197
The Coping with Death Scale Short Version	0.050	0.021	5.885	0.015	1.052	1.010–1.096

## 4 Discussion

There is heterogeneity in nurses’ the quality of palliative nursing care. This study identified two potential categories of palliative nursing care quality through latent profile analysis, “low palliative nursing care quality group” and “high palliative nursing care quality group,” indicating heterogeneity. The “low palliative nursing care quality group” accounted for 47%, indicating that some nurses’ palliative nursing care quality was at a low level. At the same time, the survey found that the PNCQS average score of nurses was (3.76 ± 0.77), which was at a low level, but higher than that of Hao Yanping (4), possibly because most nurses in this study had bachelor’s degree or above. The imperfect development (16) of palliative nursing in China, not much nurses’ learning experience and inadequate staffing of human resources in clinical work may all reduce the palliative nursing care quality. After analyzing the scores of each dimension, it was found that the dimension with the highest average score was the dimension of symptom control and remission (3.87 ± 0.81), which was similar to the results of Nguyen et al. (17). It indicates that the nurses in this study had a relatively high frequency of pain assessment and symptom control, and the quality level of symptom control was reasonable, which reflects the relatively standardized and professional practice performance of nurses in palliative care in China. The average score of the spiritual support dimension was low (3.70 ± 0.81). Spiritual support is an integral part of palliative care (18, 19), but the ability of spiritual support in Chinese nurses is low. In addition to nurses’ heavy clinical work and insufficient time to provide spiritual support to patients, the lack of knowledge about spiritual care is also an important reason for nurse’ low level of cognition and practice frequency of spiritual support. The dimension with the lowest average score was the dimension of continuity of care (3.68 ± 0.92). Palliative care emphasizes that patients should have access to appropriate medical resources and receive continuous care throughout their terminal stage. The results of this study show that Chinese nurses are insufficient in the continuity of care, which may be caused by the shortcomings of the communication and coordination mechanism among Chinese medical institutions (20). In order to improve nurses’ the quality of palliative nursing care, it is suggested to improve the training system of palliative care course, rationally plan the palliative care team and pay attention to the sustainability of care.

Influencing factors of nurses’ the quality of palliative nursing care. (1) Attitude of nurses toward palliative care. The results of this study showed that the attitude of nurses toward palliative care is an important factor affecting the palliative nursing care quality. Theory of planned behavior states that behavior is guided by a person’s attitudes and beliefs (21). Therefore, improving the attitude of nurses toward palliative care is beneficial to providing palliative care to terminal stage patients in medical institutions. The results of this study showed that the total score of the Bradley Attitude Assessment Questionnaire was (37.87 ± 3.64) points, the average score was (3.16 ± 0.30) points, which was similar to the research results of Chen et al. (22) and at a medium level. Nurses with a higher level of palliative care knowledge can better understand the significance of palliative care for terminal stage patients, and have a more positive attitude, so they are more willing to implement palliative care for terminal stage patients. Therefore, nursing managers should further strengthen the training of nurses’ professional knowledge of palliative care. After analyzing the scores of each dimension, it was found that the dimension with the lowest average score was communication dimension (2.77 ± 0.50). On the one hand, it may be due to the lack of efficient communication skills of nurses, so that they cannot provide high-quality communication for patients and their families; On the other hand, under the influence of our traditional culture, nurses may avoid discussing the topic of death with them, thus affecting the communication with patients. In the process of hospice practice in the future, nurses should periodically and systematically learn and consolidate the theoretical knowledge and practical skills of hospice communication. (2) Meaning in life. The results of this study showed that the meaning in life is an important factor affecting the palliative nursing care quality. Meaning in life means that an individual feels the value of his own life and clearly understands his own direction or mission, which is composed of the dimension of sense of existential meaning and sense of seeking meaning (9). Individuals with a high meaning in life are more able to perceive the positive feelings brought by work, relieve the negative emotions in life, and make them focus on nursing work and contribute their talents and advantages (23). The results of this study showed that the total score of the Meaning in Life Questionnaire was (45.10 ± 8.83) points, the average score was (5.01 ± 0.98) points, which was similar to the research results of Liu (24) and at a medium level. This may be due to the special working environment of nurses, who often face more deaths, which inspires nurses to realize the meaning of their own life, think about their mission in limited life, and clarify and pursue their own life goals. To further improve nurses’ meaning in life, nursing managers can, on the one hand, encourage nurses to participate in the training of hospice nurses (25), and provide direction for nurses’ career development. On the other hand, nurses are encouraged to contact mental health professionals, form a correct view of life and death, respect and cherish life, and play a personal value during life. (3) Coping with death competence. The results of this study showed that the coping with death competence is an important factor affecting the palliative nursing care quality. Coping with death competence, also known as death self-efficacy, refers to the adaptive behaviors that individuals can actively make when facing death (26). It is a series of skills and attitudes or beliefs that individuals adopt when facing death events. Coping with death competence is one of the skills a nurse must have. If nurses cannot effectively face death, it will not only affect the survival experience of patients at the last moment of life, but also easily lead to dissatisfaction of patients’ families and even medical disputes. At the same time, it will also affect the physical and mental health of nurses and produce negative emotions such as fear of death and death anxiety (27). The results of this study showed that the total score of the Coping with Death Scale Short Version was (41.91 ± 9.488) points, which was at a low level. An Australian study (28) showed that MOOC online education training significantly improved participants’ coping with death competence. To improve nurses’ coping with death competence, it is suggested that nursing managers should conduct death education training for nurses.

## 5 Conclusion

There are two latent categories of nurses’ the quality of palliative nursing care, and 47% of nurses’ palliative nursing care quality is at a low level. Attention should be paid to nurses with low attitude of nurses toward palliative care, meaning in life and coping with death competence, and precise interventions should be implemented for different categories of nurses to improve their palliative nursing care quality. In the future, longitudinal studies can be conducted to dynamically observe the changing trend of nurses’ palliative nursing care quality. We should conduct in-depth interviews to identify the influencing factors and explore intervention strategies to improve nurses’ palliative nursing care quality.

## Data Availability

The original contributions presented in this study are included in this article/supplementary material, further inquiries can be directed to the corresponding authors.

## References

[1] National Cancer Institute. *Definition of Hospice[EB/OL].[201612-20].* (2016). Available online at: http://medlineplus.gov/hospicecare.html (accessed October 16, 2016).

[2] Hagan T Xu J Lopez R Bressler T. Nursing’s role in leading palliative care: A call to action. *Nurse Educ Today.* (2018) 61:216–9. 10.1016/j.nedt.2017.11.037 29245101 PMC5859921

[3] Zulueta Egea M Prieto-Ursúa M Bermejo Toro L. Good palliative nursing care: Design and validation of the palliative nursing care quality scale (PNCQS). *J Adv Nurs.* (2020) 76:2757–67. 10.1111/jan.14464 32770576

[4] Ying Z Lan L Yanping H. Investigation and countermeasure analysis of palliative nursing care quality. *Chin Nurs Res.* (2023) 37:3981–9.

[5] Misitano A Moro A Ferro M Forresi B. The dissociative subtype of post-traumatic stress disorder: A systematic review of the literature using the latent profile analysis. *J Trauma Dissociation.* (2024) 25:349–65. 10.1080/15299732.2022.2120155 36062756

[6] Ferguson S Moore G Hull D. Finding latent groups in observed data: A primer on latent profile analysis in Mplus for applied researchers. *Int J Behav Dev.* (2019) 44:458–68. 10.1177/0165025419881721

[7] Bradley E Cicchetti D Fried T Rousseau D Johnson-Hurzeler R Kasl S Attitudes about care at the end of life among clinicians: A quick, reliable, and valid assessment instrument. *J Palliat Care.* (2000) 16:10802958. 10.1177/082585970001600103 10802958

[8] Zhou M. *Investigation and Analysis of the Status Quo of Knowledge and Attitudes about Hospice Care Among Nurses in Shanghai.* Shanghai: Second Military Medical University (2007). 10.7666/d.y1180119

[9] Steger MF Frazier P Oishi S Kaler M. The meaning in life questionnaire: Assessing the presence of and search for meaning in life. *J Counsel Psychol.* (2006) 53:80–90. 10.1037/0022-0167.53.1.80

[10] Liu S Gan Y. Reliability and validity of the Chinese version of the meaning in life questionnaire (in Chinese). *Chin Ment Health J.* (2013) 24:478–82.

[11] Galiana L Oliver A De Simone G Linzitto J Benito E Sansó NA. Brief measure for the assessment of competence in coping with death: The coping with death scale short version. *J Pain Symptom Manage.* (2019) 57:209–15. 10.1016/j.jpainsymman.2018.11.003 30447381

[12] Li Q Shi B. Chinese translation of the brief version of coping with death scale and application in oncology nurses. *Chin Nurs Res.* (2021) 35:2585–9. 10.27366/d.cnki.gtyku.2021.000783

[13] Tein J Coxe S Cham H. Statistical power to detect the correct number of classes in latent profile analysis. *Struct Equ Model.* (2013) 20:640–57. 10.1080/10705511.2013.824781 24489457 PMC3904803

[14] George D Mallery P. *IBM SPSS Statistics 25 Step by Step: A Simple Guide and Reference.* 15th ed. London: Routledge (2018). 10.4324/9781351033909

[15] Nathanson V. Revising the declaration of Helsinki. *BMJ.* (2018) 346:f2837. 10.1136/bmj.f2837 23657182

[16] Zhang YH Wei WH Yao Y. Status quo of patients’ perception of hospice care quality and its influencing factors. *Chin Nurs Manage.* (2021) 21: 1643–6.

[17] Nguyen D Lai W. Difficulties and practices in palliative nursing for cancer patients in Vietnam. *J Hosp Palliat Nurs.* (2021) 23:512–9. 10.1097/NJH.0000000000000787 34714801

[18] Park C Sacco S. Heart failure patients’ desires for spiritual care, perceived constraints, and unmet spiritual needs: relations with well-being and health-related quality of life. *Psychol Health Med.* (2017) 22:1011–20. 10.1080/13548506.2016.1257813 27838924

[19] Hu Y Liu T Li F. Association between dyadic interventions and outcomes in cancer patients: A meta-analysis. *Support Care Cancer.* (2019) 27:745–61. 10.1007/s00520-018-4556-8 30604008

[20] Su J Xu L Yi C Ding H Zuo W Guo F. Reflection on the implementation of community-based Bi-directional referrals in the construction of regional medical consortium. *Chin General Pract.* (2020) 23:1541–6. 10.12114/j.issn.1007-9572.2020.00.197

[21] Yang L Liu Y Sun H Chiang H Tsai Y Liaw J. Psychometric testing of two Chinese-version scales on attitudes toward and caregiving behaviors for end-of-life patients and families. *Clin Nurs Res.* (2018) 27:1017–40. 10.1177/1054773817699931 28347149

[22] Chen L Li XH Pan X Pan QN Huang HQ Tao PY Nurses’ knowledge, attitudes, and willingness to practice hospice care: An analysis of influencing factors. *PLoS One.* (2022) 17:e0259647. 10.1371/journal.pone.0259647 35202415 PMC8870562

[23] Zheng R Guo Q Dong F Owens R. Chinese oncology nurses’ experience on caring for dying patients who are on their final days: A qualitative study. *Int J Nurs Stud.* (2015) 52:288–96. 10.1016/j.ijnurstu.2014.09.009 25445033

[24] Liu Q Chang R Fang S Peng J. Chain mediating role of moral values identification and positive psychological capital in the relationship between meaning in life and crisis vulnerability. *Medicine.* (2024) 103:e39781. 10.1097/MD.0000000000039781 39331901 PMC11441967

[25] Zheng Z Li X. D*omestic and foreign research progress on certification and core competencies of palliative care clinical nurse specialists. *J Nurs Sci.* (2017) 32: 109–12.

[26] Chan W Tin A Wong K. Coping with existential and emotional challenges: Development and validation of the self-competence in death work scale. *J Pain Symptom Manage.* (2015) 50:99–107. 10.1016/j.jpainsymman.2015.02.012 25701687

[27] Lehto R Stein K. Death anxiety: An analysis of an evolving concept. *Res Theory Nurs Pract.* (2009) 23:23–41. 10.1891/1541-6577.23.1.23 19418886

[28] Miller-Lewis L Tieman J Rawlings D Parker D Sanderson C. Can exposure to online conversations about death and dying influence death competence? An exploratory study within an Australian massive open online course. *Omega.* (2020) 81:242–71. 10.1177/0030222818765813 29580148

